# Effects of local structural transformation of lipid-like compounds on delivery of messenger RNA

**DOI:** 10.1038/srep22137

**Published:** 2016-02-26

**Authors:** Bin Li, Xiao Luo, Binbin Deng, JoLynn B. Giancola, David W. McComb, Thomas D. Schmittgen, Yizhou Dong

**Affiliations:** 1 Division of Pharmaceutics and Pharmaceutical Chemistry, College of Pharmacy, The Ohio State University, Columbus, Ohio 43210, USA; 2 Center for Electron Microscopy and Analysis, Department of Materials Science and Engineering, The Ohio State University, Columbus, Ohio 43212, USA; 3 Department of Chemistry and Biochemistry, The Ohio State University, Columbus, Ohio 43210, USA; 4 Division of Pharmaceutics, College of Pharmacy, University of Florida, Gainesville, Florida 32610, USA

## Abstract

Lipid-like nanoparticles (LLNs) have shown great potential for RNA delivery. Lipid-like compounds are key components in LLNs. In this study, we investigated the effects of local structural transformation of lipid-like compounds on delivery of messenger RNA. Our results showed that position change of functional groups on lipid-like compounds can dramatically improve delivery efficiency. We then optimized formulation ratios of TNT-b_10_ LLNs, a lead material, increasing delivery efficiency over 2-fold. More importantly, pegylated TNT-b_10_ LLNs is stable for over four weeks and is over 10-fold more efficient than that of its counterpart TNT-a_10_ LLNs. Additionally, the optimal formulation O-TNT-b_10_ LLNs is capable of delivering mRNA encoding luciferase *in vivo*. These results provide useful insights into the design of next generation LLNs for mRNA delivery.

Lipid-like nanoparticles (LLNs, termed Lipidoids) are a structurally diverse library of lipid-like compounds formulated materials. Previous studies demonstrated that lipid-like nanoparticles (LLNs) are suitable for delivery of siRNA and mRNA both *in vitro* and *in vivo*^1^,^2^,^3^,^4^,^5^,^6^,^7^,^8^. Moreover, lead LLNs show a broad therapeutic window and are promise for therapeutic applications^2^,^3^,^9^. LLNs are normally formulated with lipid-like compounds, phospholipids, cholesterol, and polyethylene glycol derivatives^1^,^10^. Although each component is necessary to form stable nanoparticle formulations, lipid-like compounds, consisting of amino groups and multiple lipid tails, play a significant role for efficient delivery of RNA^11^. Using a combinatorial library strategy, a wide variety of lipid-like compounds have been developed^1^,^2^,^12^. Among these lipid-like compounds, amino alcohol-based lipids displayed superior activity^2^. However, little studies have explored the effects of local structural transformation of lipid-like compounds on delivery efficiency^13^,^14^.

We previously reported a series of lipid-like 1,3,5-triazinane-2,4,6-trione (TNT) derivatives consisting of a six-membered ring and three lipid tails, among of which TNT-a_10_ shows efficient delivery of siRNA (Fig. 1B)^15^. In order to investigate the effects of local structural transformation of lipid-like compounds on messenger RNA delivery, herein we report the synthesis of lipid-like compounds TNT-b_8_ to TNT-b_14_ and their delivery efficiency of mRNA (Fig. 1A). TNT-b_8_ to TNT-b_14_ possess the same six-member ring structure as previously reported TNTs: 1,3,5-triazinane-2,4,6-trione, while the position of hydroxyl and amino groups is exchanged (Fig. 1B). After formulation, TNT-b_10_ LLNs showed higher delivery efficiency of mRNA encoding firefly luciferase (FLuc) in comparison to other TNT LLNs. Further optimization of formulation ratios improved the efficiency of TNT-b_10_ LLNs over two fold, which was more than 10-fold more potent than TNT-a_10_ LLNs formulated under the same condition. Lastly, we studied delivery efficiency of the optimized TNT-b_10_ LLNs (O-TNT-b_10_ LLNs) *in vivo* through three administration routes including intravenous (i.v.), intraperitoneal (i.p.), and subcutaneous (s.c.).

## Results

**Figure 1:**
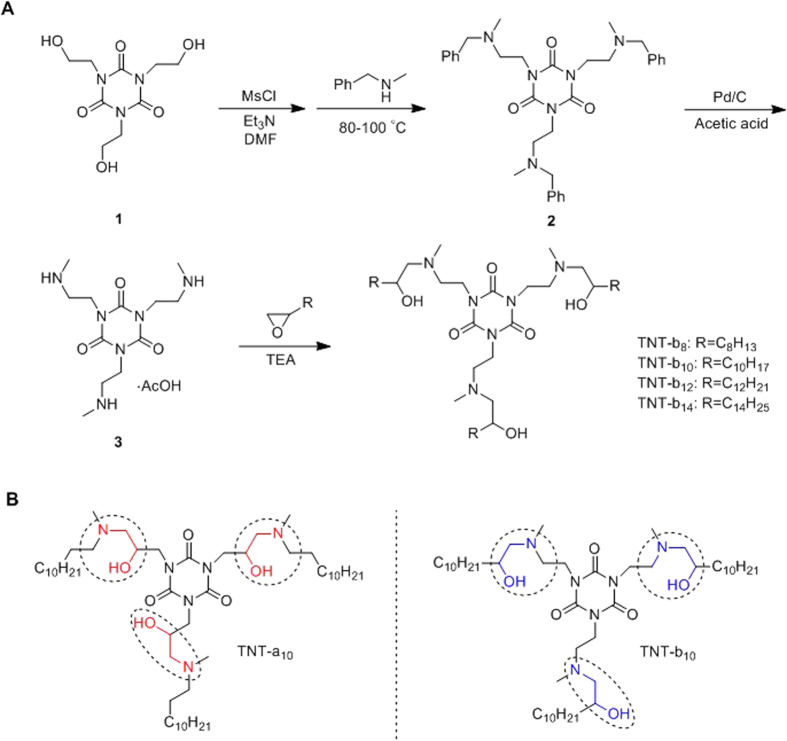
(**A**) Synthesis of lipid-like compounds 1,3,5-triazinane-2,4,6-triones derivatives (TNT-b_8_ to TNT-b_14_). (**B**) Structural comparison of TNT-a_10_ and TNT-b_10_. Compared to the structure of TNT-a_10_, a previously reported lipid-like compound, the positions of hydroxyl and amino groups were exchanged in the structure of TNT-b_10_.

Non-viral drug delivery systems have shown great potential for diagnostic and therapeutic applications^6^,^14^,^16^,^17^,^18^,^19^,^20^,^21^,^22^,^23^,^24^. Among the wide variety of these systems, lipid- and polymer-based nanomaterials have been reported for mRNA delivery^25^,^26^,^27^,^28^,^29^,^30^. However, our knowledge into the structure-activity relationship remains limited. In order to investigate the effects of structural transformation of lipid-like compounds on mRNA delivery efficiency, we designed and synthesized TNT-b_8_ to TNT-b_14_. First, compound **1** was reacted with MsCl and subsequently N-methyl-1-phenylmethanamine to produce compound **2**, hydrogenation of which in the presence of Pd/C afforded compound **3**^31^. Compound **3** underwent a ring-opening reaction with epoxides to yield TNT-b_8_ to TNT-b_14_^3^, composed of a six-member ring core (1,3,5-triazinane-2,4,6-trione) and three amino lipid chains (from C8 to C14, Fig. 1A). Structures of TNTs were confirmed by ^1^H NMR and MS (Supporting information).

**Figure 2:**
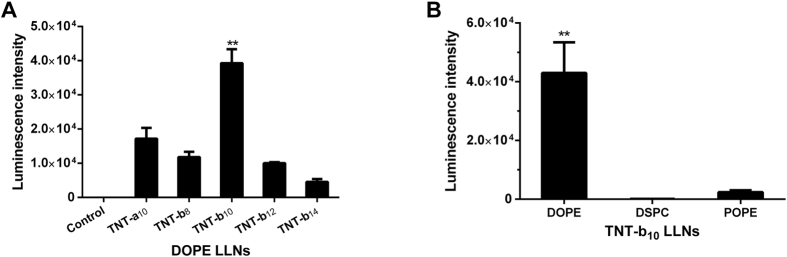
*In vitro* mRNA delivery of TNT LLNs in Hep 3B cells. (**A**) TNT-b_10_ LLNs showed higher transfection efficiency than TNT-a_10_, TNT-b_8_, TNT-b_12_, and TNT-b_14_ LLNs. **p < 0.01 as determined by an unpaired student’s t-test. These results indicate that not only the length of carbon chains but also the position of functional groups of lipid-like compounds have dramatic effects on delivery efficiency. (**B**) The effect of phospholipids on LLNs-mediated transfection. DOPE-formulated LLNs were more efficient for mRNA delivery compared to DSPC and POPE-formulated LLNs. **p < 0.01 as determined by an unpaired student’s t-test.

In order to investigate the delivery efficiency of TNT-a_10_ and TNT-b_8_ to TNT-b_14_, we utilized a luciferase expression assay in Hep 3B cells, a human hepatoma cell line^5^. TNTs were formulated with DOPE, cholesterol, DMG-PEG_2000_ and Fluc mRNA using the previously optimized formulation ratio (TNTs/DOPE/Cholesterol/DMG-PEG_2000_ = 20/30/40/0, Fig. 2). Cells were treated with freshly formulated TNT LLNs for 6 h and luciferase activity was then quantified. As shown in Fig. 2A, TNT-b_10_ LLNs showed higher transfection efficiency than TNT-b_8_, TNT-b_12_, and TNT-b_14_ LLNs. Moreover, TNT-b_10_ LLNs was over 2-fold more efficient compared to TNT-a_10_, structure of which is similar to that of TNT-b_10_ except the position exchange of hydroxyl and amino groups. These results indicate that not only the length of carbon chains but also the position of functional groups of lipid-like compounds have dramatic effects on delivery efficiency.

In order to evaluate the effects of phospholipids, we next formulated TNT-b_10_ with DSPC or POPE, another two widely used phospholipids in nanoparticle formulations. Consistent with previous studies^5^,^6^, DOPE-formulated LLNs were more efficient for mRNA delivery compared to DSPC and POPE-formulated LLNs (Fig. 2B). Consequently, DOPE was selected for further *in vitro* optimization of TNT-b_10_ LLNs.

**Figure 3:**
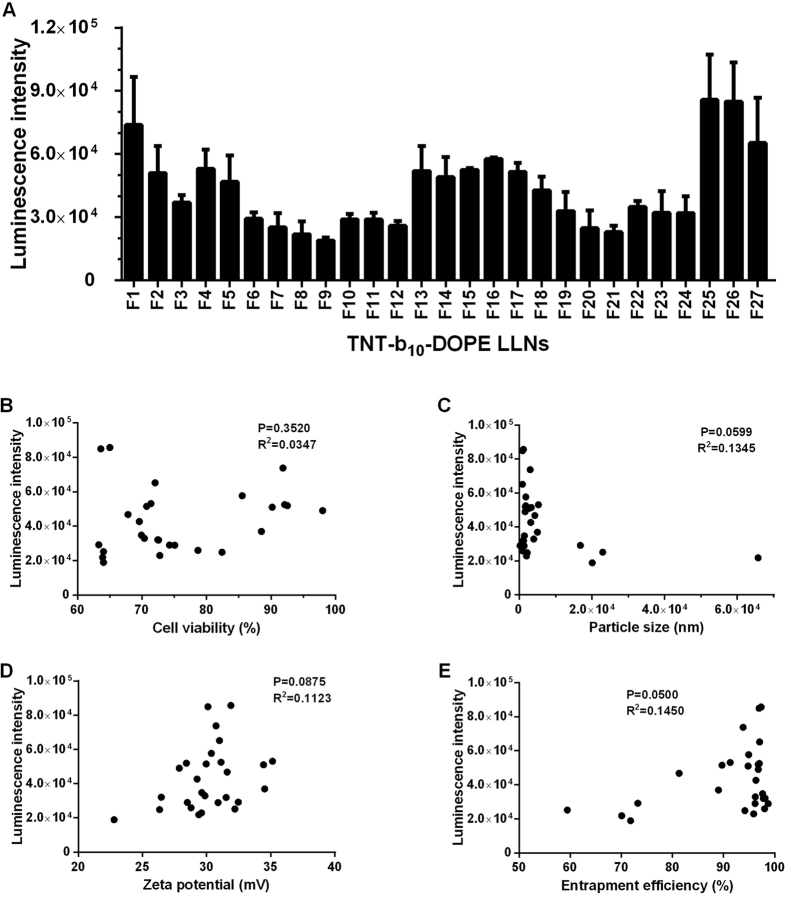
(**A**) Optimization of TNT-b_10_ LLNs. Formulation 25 (F25) showed the highest luciferase expression in Hep 3B cells at a dose of 200 ng of luciferase mRNA. (**B–E**) Correlation analysis between transfection efficiency and cell viability, particle size, zeta potential, and entrapment efficiency. Positive correlation between luciferase intensity and entrapment efficiency was observed, while no significant correlation was found between luciferase intensity and particle size, zeta potential, or cell viability.

Previously, we utilized an orthogonal experimental design to optimize the formulation ratio, which has been demonstrated as a useful tool^5^. In this study, we assigned three levels for each component, which provide 27 formulations (Supporting information, Table S1 and S2). Formulation 25 (F25) with a combination of TNT-b10/DOPE/Cholesterol = 30/40/35 was 2-fold more efficient compared to the initial formulation (TNT-b10/DOPE/Cholesterol = 20/30/40) (Fig. 3A). We then performed a correlation analysis of particle properties and delivery efficiency. We observed positive correlation between luciferase intensity and entrapment efficiency, while no significant correlation was found between luciferase intensity and particle size, zeta potential, or cell viability (Fig. 3B–E).

**Figure 4:**
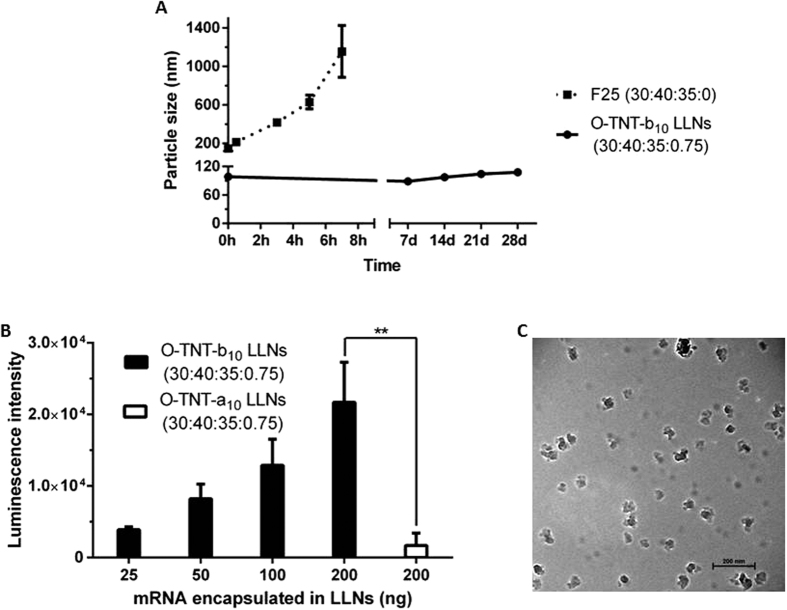
(**A**) Effects of pegylation on particle stability. After pegylation, O-TNT-b_10_ LLNs was stable for at least four weeks. (**B**) O-TNT-b_10_ LLNs-mediated dose-dependent expression of luciferase in Hep 3B cells. Delivery efficiency of O-TNT-b_10_ LLNs was 10-fold higher than that of TNT-a_10_ LLNs formulated with the same ratio. (**C**) A Cryo-EM image of O-TNT-b_10_ LLNs (Scale bar, 200 nm). **p < 0.01 as determined by an unpaired student’s t-test.

Yet, we observed that the particle size of F25 increased significantly several hours after formulation (Fig. 4A). After incorporation of DMG-PEG_2000_ [TNT-b10/DOPE/Cholesterol/DMG-PEG_2000_ = 30/40/35/0.75; termed optimized TNT-b_10_ LLNs (O-TNT-b_10_ LLNs)] according to previous results^5^, the particles were stable for at least four weeks (Fig. 4A). Consistent with previous reports^5^,^32^,^33^, pegylation improved particle stability while hindered transfection efficiency of LLNs: F25 without pegylation showed higher luciferase intensity than O-TNT-b_10_ LLNs *in vitro* (Supporting information, Fig S1). We then evaluated the delivery efficiency of O-TNT-b_10_ LLNs at four different doses: 25, 50, 100, and 200 ng/well. As shown in Fig. 4B, O-TNT-b_10_ LLNs displayed dose-dependent expression of luciferase *in vitro*. More importantly, delivery efficiency of O-TNT-b_10_ LLNs was 10-fold higher than that of TNT-a_10_ LLNs formulated under the same condition (Fig. 4B, **p < 0.01). A Cryo-TEM image illustrated that O-TNT-b_10_ LLNs formed irregular nanoparticles with particle size consistent with the results from dynamic light scattering (Figs 4A-4C).

**Figure 5:**
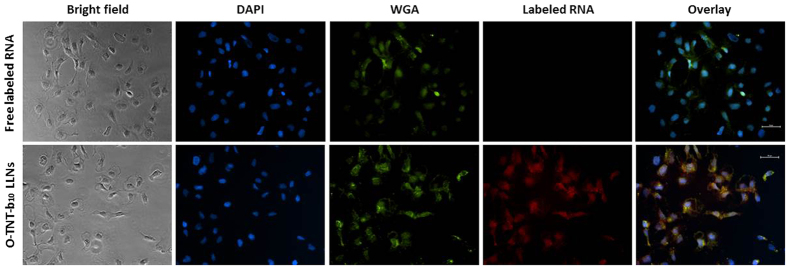
Fluorescence imaging of Hep 3B cells treated with O-TNT-b_10_ LLNs containing labeled RNA (red). Cells were stained with DAPI (blue, nuclei) and WGA (green, cell membranes). Free labeled RNA served as a control. Scale bar, 50 μm.

In order to visualize cellular uptake of O-TNT-b_10_ LLNs, we treated cells with O-TNT-b_10_ LLNs encapsulated Alexa-Fluor 647 labeled RNA (red). 3 h after treatment, Hep 3B cells were fixed with formaldehyde. Nuclei and membrane were then stained with DAPI (blue) and Alexa-Fluor 488 conjugate of wheat germ agglutinin (green), respectively. A dramatic cellular uptake of O-TNT-b_10_ LLNs was detected using fluorescence microscopy compared to a control group treated with free labeled RNA (Figs 5).

**Figure 6:**
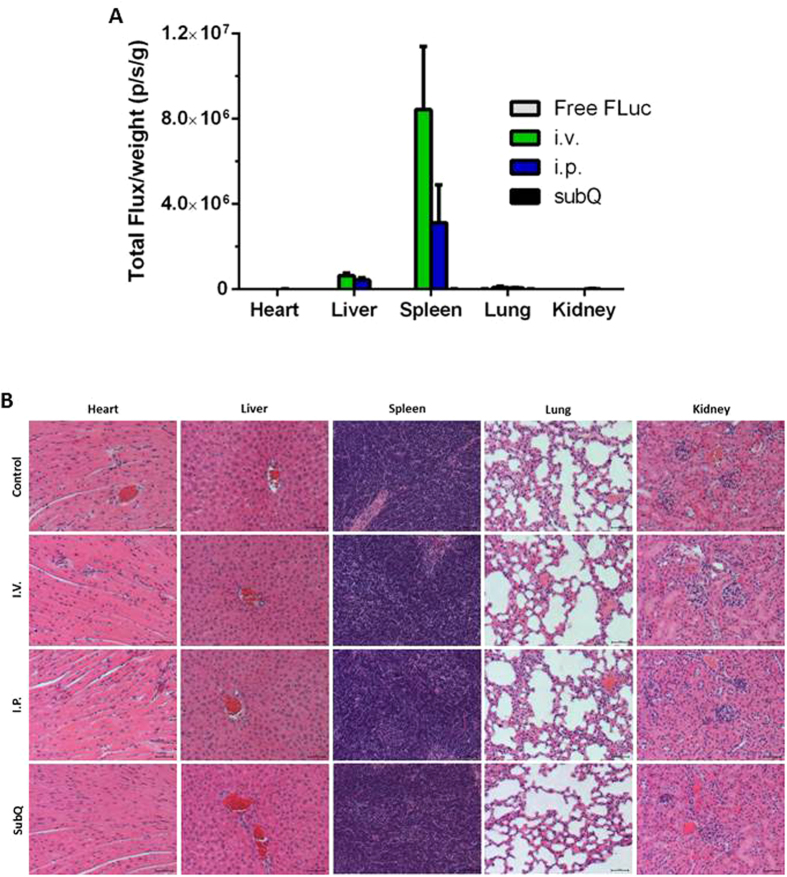
(**A**) Delivery of mRNA encoding luciferase using O-TNT-b_10_ LLNs in mice. O-TNT-b_10_ LLNs (0.5 mg/kg) were administered in mice via three injection routes: intravenous (i.v.), intraperitoneal (i.p.), and subcutaneous (s.c.). Six hours after injection, the luminescence signal was quantified via a Xenogen IVIS imaging system. Data are presented as total flux normalized by tissue weight. (**B**) Histopathological analysis of tissues. The untreated group served as a control. Scale bar, 50 μm.

Lastly, in order to study delivery efficiency of O-TNT-b_10_ LLNs *in vivo*, O-TNT-b_10_ LLNs were administered in mice via three injection routes: intravenous (i.v.), intraperitoneal (i.p.), and subcutaneous (s.c.). Intravenous and intraperitoneal injections of O-TNT-b_10_ LLNs showed high expression of luciferase in the liver and spleen. No detectable signal was observed in kidney, heart, and lung. No signal was detected in mice treated with subcutaneous injection of O-TNT-b_10_ LLNs and intravenous injection of free mRNA. Interestingly, a significantly higher signal was detected in the spleen compared to the liver (over 10-fold) in mice with intravenous injections of O-TNT-b_10_ LLNs (Fig. 6A). These results demonstrated that O-TNT-b_10_ LLNs is capable of delivering mRNA *in vivo* and shows a unique expression in the spleen may have potential therapeutic applications for spleen disorders. A preliminary histology analysis indicated no significant pathological changes in all treated groups compared with the control group (Fig. 6B).

## Discussion

In conclusion, we designed and synthesized TNT-b_8_ to TNT-b_14_ in order to investigate the effects of local structural transformation on mRNA delivery. TNT-b_8_ to TNT-b_14_ were composed of a phenyl ring, three amide linkers, and three amino lipid tails. Compared to the structure of TNT-a_10_, a previously reported lipid-like compound, TNT-b_8_ to TNT-b_14_ exchanged the positions of hydroxyl and amino groups. According to an *in vitro* luciferase assay, TNT-b_10_ LLNs were 2-fold more efficient than TNT-a_10_ LLNs, demonstrating the importance of local structural transformations. The correlation analysis of delivery efficiency and particle properties showed a positive correlation between delivery efficiency and mRNA entrapment percentage, consistent with our results reported previously^5^. Optimization of formulation ratios improved delivery efficiency over 2-fold. Because pegylation stabilized the LLNs, we identified an optimal formulation O-TNT-b_10_ LLNs, 10-fold more efficient compared to TNT-a_10_ LLNs formulated with the same formulation ratios. Cellular uptake of O-TNT-b_10_ LLNs was visualized by fluorescence imaging analysis. More importantly, O-TNT-b_10_ LLNs is capable of delivering mRNA encoding luciferase through intravenous and intraperitoneal administration, but not subcutaneous administration. Interestingly, we observed a substantially higher expression in the spleen compared to other organs including the liver. No obvious toxicity was detected from histological analysis. Reflecting the results all above, O-TNT-b_10_ LLNs merit further development for treating spleen disorders.

## Methods

### Materials

N-methyl-1-phenylmethanamine, Et_3_N, Pd/C, epoxides and other chemicals were purchased from Sigma-Aldrich. 1,2-distearoyl- sn-glycero-3-phosphocholine (DSPC), 1,2-dioleoyl-sn-glycero-3-phosphoethanolamine (DOPE), and 1-palmitoyl-2-oleoyl- sn-glycero-3-phosphoethanolamine (POPE) were purchased from Avanti Polar Lipids, Inc. 3-(4,5-dimethylthiazol-2-yl)-2,5-diphenyltetrazolium bromide (MTT) was purchased from Amresco (Solon, OH). Buffered formaldehyde (10%, pH 7.4) was purchased from Ricca Chemical (Arlington, TX). Firefly luciferase mRNA (FLuc mRNA) was purchased from TriLink Biotechnologies, Inc. (San Diego, CA). Alexa fluor 488 conjugate of wheat germ agglutinin, NucBlue Fixed cell ready probes DAPI, ProLong diamond antifade mountant reagent, Ribogreen reagent and fetal bovine serum (FBS) were purchased from Life Technologies (Grand Island, NY). Alexa-Fluor 647-labeled RNA was purchased from Integrated DNA Technologies. Bright-Glo luciferase assay substrate was from Promega (Madison, WI, USA).

### Synthesis of lipid-like materials TNTs

Compounds **1**–**3** were synthesized according to the literature^31^. To a solution of compound **1** (5 g) and triethylamine (Et_3_N, 10 mL) in DMF (20 mL) was added methanesulfonyl chloride (MsCl, 5 mL) dropwise. After reaction, the mixture was poured into ice-water (100 mL), filtered and dried overnight. Then the intermediate was reacted with excessive N-methyl-1-phenylmethanamine (20 g) overnight at 90 °C. The reaction mixture was purified using silica gel chromatography to obtain compound **2**. The resulting compound **2** was hydrogenated at 130 psi in the presence of Pd/C at RT to give compound **3**. Compound **3** (0.2 mmol), epoxide (0.9 mmol), and TEA (0.8 mmol) were mixed and stirred at RT for 30 min, then reacted at 150 °C under microwave for 5 h. The mixture was purified by silica gel chromatography to afford TNTs. Structures of TNTs were confirmed by ^1^H NMR spectra (400 MHz, Bruker) and mass spectrometer (Micromass Q-TOF micro).

### Preparation and characterization of LLNs encapsulated FLuc mRNA

TNT LLNs were formulated with TNTs, phospholipid (DOPE, DSPC or POPE), cholesterol and FLuc mRNA as reported previously^5^. Briefly, TNT, phospholipid, and cholesterol were dissolved in ethanol at a molar ratio of 20:30:40, and mRNA was dissolved in 10 mM sodium acetate buffer (pH = 3).The ethanol phase was then mixed well with equal volume of mRNA solution (the weight ratio of TNT: mRNA = 10: 1). Finally, the formulated LLNs were diluted by equal volume of PBS. For *in vivo* experiments, LLNs were formulated using a microfluidic-based mixing device (Precision Nanosystems). The particle size of formulations was determined by dynamic light scattering using a Zetasizer NanoZS (Malvern, Worcestershire, UK) at a scattering angle of 173°. The encapsulation efficiency of mRNA in LLNs was quantified by a RiboGreen assay.

### Cell transfection and formulation Optimization

Hep 3B cells purchased from American Type Culture Collection (Manassas, VA) were seeded into 96-well opaque white plates in Eagle’s Minimum Essential Medium (EMEM) supplemented with 10% heat inactivated FBS at a density of 20,000 cells per well. After overnight incubation, cells were transfected by LLNs containing 200 ng of FLuc mRNA for 6 h. The culture medium containing formulations was then discarded, and luciferase activity was quantified using a SpectraMax M5 microplate reader after adding 50 μL of serum-free EMEM and 50 μL of Bright-Glo luciferase reagent (Promega). In order to optimize formulation ratio of TNT-b_10_ LLNs, 27 formulations prepared using the ratio listed in Supplementary Table S1 were evaluated with the above-mentioned method. Cells treated with free FLuc mRNA were used as a control group. Transfections were performed in triplicate.

### Cytotoxicity assay

The cytotoxicity of formulations against Hep3B cells was determined using an MTT assay^5^. Hep 3B cells were seeded in 96-well clear plates (20,000 cells/well) overnight and were then treated with LLNs (equivalent to 200 ng of FLuc mRNA). After 6 h treatment, MTT was added and incubated for another 4 h. The medium was then removed, and 150 μL of dimethylsulfoxide was added. Ten minutes later, the absorbance at a wavelength of 570 nm was measured on a SpectraMax M5 microplate reader (Molecular Devices, Sunnyvale, CA). Cell viability was normalized by untreated cells.

### Cryo-transmission electron microscopy

A small volume (3–5 μL) of samples was added to a lacey carbon coated 300 mesh copper grid. The grid was blotted and immediately plunged into liquid ethane to rapidly form a thin film of amorphous ice and then transferred to a Gatan 626 cryo-transfer holder (Gatan, Pleasanton, CA). Cryo-transmission electron microscopy (Cryo-TEM) images were taken at low dose conditions in a Tecnai F20 S/TEM (FEI, Hillsboro, OR). The frozen grids were kept under liquid nitrogen temperature at all times.

### Cellular uptake

Hep3B cells were seeded in a sterile 6-well plate and grown to about 70% confluent prior to treatment. Cells were then treated with free labeled RNA or O-TNT-b_10_ LLNs containing labeled RNA (the final concentration of labeled RNA is 0.25 μg/mL). After 3 h treatment, cells were rinsed and fixed by 4% formaldehyde for 10 min. Cells were then washed three times and stained with DAPI and Alexa-Fluor 488 conjugate of wheat germ agglutinin (final concentration of 1 μg/mL). Cells were washed three times again and mounted onto slides using a ProLong diamond antifade mountant reagent. Images were taken using an ECLIPSE Ti inverted fluorescence microscopy (Nikon, Japan).

### mRNA delivery *in vivo* and histopathological analysis

All procedures in animal studies conducted at The Ohio State University were approved by the Institutional Animal Care and Use Committee (IACUC) and were consistent with local, state and federal regulations as applicable. C57BL/6 mice (3 mice/per group) were injected free FLuc mRNA or FLuc mRNA encapsulated O-TNT-b_10_ LLNs at a dose of 0.5 mg/kg via three administration routes (i.v., i.p. and s.c.)^34^. Six hours after administration, D-luciferin substrate (150 μL, 30 mg/mL) were then i.p. injected into the mice. Eight minutes later, mice were sacrificed and organs including heart, liver, spleen, lung and kidney were harvested. The luminescence was immediately measured by a Xenogen IVIS imaging system (Caliper, Alameda, CA) and normalized against organ weight. For histopathological analysis, organs were fixed overnight in 10% formaldehyde and transferred to 70% ethanol. After paraffin embedding, sectioning and hematoxylin and eosin staining, histopathological examination was conducted using an ECLIPSE Ti inverted fluorescence microscopy (Nikon, Japan).

## Additional Information

**How to cite this article**: Li, B. *et al.* Effects of local structural transformation of lipid-like compounds on delivery of messenger RNA. *Sci. Rep.* **6**, 22137; doi: 10.1038/srep22137 (2016).

## Supplementary Material

Supplementary Information
