# Supplementary material for: Effects of local structural transformation of lipid-like compounds on delivery of messenger RNA
Source: Sci Rep. 2016 Feb 26;6:22137. doi: 10.1038/srep22137 (PMC4768262; doi:10.1038/srep22137)
Supplement: Supplementary Information [file srep22137-s1.pdf]

Effects of local structural transformation of lipid-like compounds on delivery of messenger RNA

Bin Li[1,+], Xiao Luo[1,+], Binbin Deng[2], JoLynn B. Giancola[3], David W. McComb[2], Thomas D. Schmittgen[4], and Yizhou Dong[1,*]

[1]Division of Pharmaceutics and Pharmaceutical Chemistry, College of Pharmacy, The Ohio State University, Columbus, Ohio 43210, USA
[2]Center for Electron Microscopy and Analysis, Department of Materials Science and Engineering, The Ohio State University, Columbus, Ohio 43212, USA
[3]Department of Chemistry and Biochemistry, The Ohio State University, Columbus, Ohio 43210, USA
[4]Division of Pharmaceutics, College of Pharmacy, University of Florida, Gainesville, Florida 32610, USA
[*]dong.525@osu.edu
[+]these authors contributed equally to this work

Spectral data:

^{1}H NMR spectra were recorded at 300 or 400 MHz on the Bruker instrument. ^{1}H NMR chemical shifts were reported as δ values in ppm relative to TMS. Mass spectra were obtained on a Micromass Q-TOF micro Mass Spectrometer.

1,3,5-tris(2-((2-hydroxydecyl)(methyl)amino)ethyl)-1,3,5-triazinane-2,4,6-trione (TNT-b_8): yield (25%). ^{1}H NMR (300 MHz, $CDCl_3$, δ) 4.07-4.01 (3H, m), 3.96-3.93 (3H, m), 3.54 (3H, m), 3.40-3.35 (3H, m), 2.85-2.78 (3H, m), 2.58-2.53 (3H, m), 2.33-2.25 (12H, m), 1.45-1.26 (42H, m), 0.86 (9H, tri, *J* = 6.9 Hz). MS (*m/z*): $[M + H]^+$ calcd. for $C_{42}H_{85}N_6O_6$, 770; found 770.

1,3,5-tris(2-((2-hydroxydodecyl)(methyl)amino)ethyl)-1,3,5-triazinane-2,4,6-trione (TNT-b_{10}): yield (33%). ^{1}H NMR (300 MHz, $CDCl_3$, δ) 4.09-4.05 (3H, m), 3.95-3.80 (3H, m), 3.65-3.54 (3H, m), 3.39-3.35 (3H, m), 2.84-2.80 (3H, m), 2.64-2.46 (3H, m), 2.33-2.28 (12H, m), 1.53-1.03 (54H, m), 0.88 (9H, tri, *J* = 6.9 Hz). MS (*m/z*): $[M + H]^+$ calcd. for $C_{48}H_{97}N_6O_6$, 854; found 854.

1,3,5-tris(2-((2-hydroxytetradecyl)(methyl)amino)ethyl)-1,3,5-triazinane-2,4,6-trione (TNT-b_{12}): yield (39%). ^{1}H NMR (300 MHz, $CDCl_3$, δ) 4.09-4.05 (3H, m), 3.96-3.81 (3H, m), 3.54 (3H, br), 3.40-3.36 (3H, m), 2.85-2.78 (3H, m), 2.59-2.55 (3H, m), 2.32-2.28 (12H, m), 1.45-1.02 (66H, m), 0.88 (9H, tri, J = 6.9 Hz). MS (*m/z*): $[M + H]^+$ calcd. for $C_{54}H_{105}N_6O_6$, 938; found 938.

1,3,5-tris(2-((2-hydroxyoctyl)(methyl)amino)ethyl)-1,3,5-triazinane-2,4,6-trione(TNT-b_{14}): yield (42%). ^{1}H NMR (300 MHz, $CDCl_3$, δ) 4.10-3.90 (3H, m), 3.85-3.81 (6H, m), 3.54-3.36 (6H, m), 2.84-2.79 (3H, m), 2.67-2.46 (3H, m), 2.33 (9H, s), 1.44-1.25 (78H, m), 0.88 (9H, tri, *J* = 6.9 Hz). MS (*m/z*): $[M + H]^+$ calcd. for $C_{36}H_{73}N_6O_6$, 1022; found 1022.

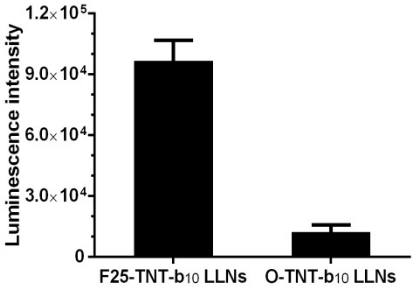

Figure S1. F25 (TNTs/DOPE/Chol = 30/40/35) without pegylation showed higher luciferase intensity than O-TNT-b_{10} LLNs *in vitro*.

Table S1. Three levels for each component: TNT-b_{10}, DOPE and Cholesterol.

Level	Formulation components (molar ratio)		
	TNT-b_{10}	DOPE	Cholesterol
1	10	20	35
2	20	30	40
3	30	40	45

Table S2. The set of formulations derived from random combinations of each level of formulation components.

Formulation	Components (molar ratio)		
	TNT-b_{10}	DOPE	Cholesterol
F1	10	20	35
F2	10	20	40
F3	10	20	45
F4	10	30	35
F5	10	30	40
F6	10	30	45
F7	10	40	35
F8	10	40	40
F9	10	40	45
F10	20	20	35
F11	20	20	40
F12	20	20	45
F13	20	30	35
F14	20	30	40
F15	20	30	45
F16	20	40	35
F17	20	40	40
F18	20	40	45
F19	30	20	35
F20	30	20	40
F21	30	20	45
F22	30	30	35
F23	30	30	40
F24	30	30	45
F25	30	40	35
F26	30	40	40
F27	30	40	45
